# RYR2 deficient human model identifies calcium handling and metabolic dysfunction impacting pharmacological responses

**DOI:** 10.3389/fcvm.2024.1357315

**Published:** 2024-07-08

**Authors:** Linda Starnes, Andrew Hall, Damla Etal, Anna-Lina Cavallo, Piotr Grabowski, John Gallon, Michelle Kha, Ryan Hicks, Amy Pointon

**Affiliations:** ^1^ Safety Sciences, Clinical Pharmacology & Safety Sciences, R&D, AstraZeneca, Gothenburg, Sweden; ^2^ Safety Sciences, Clinical Pharmacology & Safety Sciences, R&D, AstraZeneca, Cambridge, United Kingdom; ^3^ Discovery Sciences, R&D, AstraZeneca, Gothenburg, Sweden; ^4^ Imaging and Data Analytics, Clinical Pharmacology & Safety Sciences, R&D, AstraZeneca, Cambridge, United Kingdom; ^5^ BioPharmaceuticals R&D Cell Therapy Department, Research and Early Development, Cardiovascular, Renal, and Metabolism (CVRM), BioPharmaceuticals R&D, AstraZeneca, Gothenburg, Sweden; ^6^ School of Cardiovascular and Metabolic Medicine & Sciences, King’s College London, London, United Kingdom

**Keywords:** hiPSC-CMs, calcium handling, ryanodine receptor 2, pentose phosphate pathway, cardiovascular *in vitro* models, heart failure

## Abstract

Creation of disease models utilizing hiPSCs in combination with CRISPR/Cas9 gene editing enable mechanistic insights into differential pharmacological responses. This allows translation of efficacy and safety findings from a healthy to a diseased state and provides a means to predict clinical outcome sooner during drug discovery. Calcium handling disturbances including reduced expression levels of the type 2 ryanodine receptor (RYR2) are linked to cardiac dysfunction; here we have created a RYR2 deficient human cardiomyocyte model that mimics some aspects of heart failure. RYR2 deficient cardiomyocytes show differential pharmacological responses to L-type channel calcium inhibitors. Phenotypic and proteomic characterization reveal novel molecular insights with altered expression of structural proteins including CSRP3, SLMAP, and metabolic changes including upregulation of the pentose phosphate pathway and increased sensitivity to redox alterations. This genetically engineered *in vitro* cardiovascular model of RYR2 deficiency supports the study of pharmacological responses in the context of calcium handling and metabolic dysfunction enabling translation of drug responses from healthy to perturbed cellular states.

## Introduction

Translating efficacy and safety findings from healthy to diseased states is a major challenge faced during drug development (1). Patient-centric models are beginning to fill this void by providing a means to predict clinical outcome with increased accuracy and timeliness in drug discovery. The advent of induced pluripotent stem cell (iPSC) technology allows modelling of aspects of disease and drug testing *in vitro*, reducing the reliance on pre-clinical *in vivo* models and enabling a human-relevant approach (2). hiPSC-derived *in vitro* cardiovascular models that incorporate patient characteristics such as previous drug treatments, disease-related genetic mutations, age, sex and ethnicity can enable efficacy testing of novel drug candidates and prediction of drug-induced cardiotoxicity (3, 4). Such models can be developed from patient derived iPSCs and can be attenuated pharmacologically or genetically utilizing CRISPR/Cas9 technology. Disruption of calcium handling is a common theme among cardiomyopathies (5) and drug-induced cardiotoxicity (6), with indication of decreased expression of RYR2 in some forms of human heart disease (7, 8) and animal models of heart failure (9–11), implicating RYR2 dysfunction as an element of heart disease or within predisposed conditions. In order to create a broadly representative model we focused on using CRISPR/Cas9 to genetically reduce the expression level of the type 2 ryanodine receptor (RYR2).

Located within the sarcoplasmic reticulum (SR) of cardiomyocytes, the RYR2 plays a central role in calcium release (12). During the cardiomyocyte action potential, depolarization of the membrane causes opening of the L-type calcium channels (LTCC), allowing a small amount of calcium to enter the cytosol triggering a larger calcium release via RYR2 channels from the SR to the cytosol, a phenomenon known as calcium-induced calcium release (12). Calcium released into the cytosol binds to troponin C and facilitates actin-myosin cross-bridge formation and contraction. Cytosolic calcium is rapidly extruded from the cytosol back into the SR by ATP2A2 ATPase and NCX exchangers on the plasma membrane, effectively restoring intracellular calcium levels to normal resting levels allowing cardiomyocyte relaxation. Given the important role in calcium handling, dysfunction of RYR2 has been noted in several cardiac disorders. A large number of genetic mutations or abnormal post-translational modifications of the channel have been associated with catecholaminergic polymorphic ventricular tachycardia (CPVT), atrial and ventricular fibrillation, hypertrophic, dilated cardiomyopathies and sudden cardiac arrest. It is thought that the different mutations and post-translational modifications of RYR2 result in modifications of the channel activation or inactivation, that leads to enhanced or suppressed activity, respectively (13). Changes in RYR2 protein levels have also been noted in the pathogenesis of cardiac diseases (14–18). Reductions in RYR2 protein has shown enhanced susceptibility to cardiac alternans, hypertrophy and sudden death (19). Conditional cardiomyocyte specific knock-out (KO) of *Ryr2* leads to rapid onset of heart failure and sudden death (20). A more patho-physiologically relevant model, conditional deletion of one *Ryr2* allele resulting in 50% loss of RYR2 protein in adult cardiomyocytes, shows metabolic defects akin to the failing hearts, indicating that RYR2 plays a critical role in stimulating glucose oxidation *in vivo* (21). Deficiency in *Ryr2* has also been recently linked to ER stress induction (22).

Whilst there are mouse genetic models available to study RYR2 dysfunction, there are translational questions that arise as there are underlying differences compared to human in relation to heart rate, calcium handling properties and ion channel expression (23). One such human-relevant model includes utilizing human cardiomyocytes derived from induced pluripotent stem cells (hiPSC-CMs), together with CRISPR/Cas9 gene editing to introduce a RYR2-F2483I mutation. Cardiomyocytes containing the introduced RYR2-F2483I mutation showed a similar dysfunctional calcium signaling phenotype to cardiomyocytes derived from a patient biopsy carrying a F2483I mutation (24). This gives confidence that RYR2-signaling mechanisms can be studied utilizing hiPSC-CMs. Here a CRISPR/Cas9 gene editing approach was taken to study the pathophysiological consequence of reduced RYR2 protein expression in human iPSC-CMs.

## Materials and methods

### hiPSC cell culture and CRISPR/Cas9 gene editing

RIPS-1J hiPSCs were utilized for gene editing to generate *RYR2* heterozygous knockout (*RYR2* Het KO) lines. RIPS-1J hiPSCs were generated from newborn foreskin fibroblasts (BJ cells from ATCC® CRL-2522™) as described in Sjogren et al. (25). The hiPSCs were cultured in Cellartis® DEF-CS™ 500 culture system (TaKaRa) according to manufacturer's instructions. hiPSCs were grown in 6 well plates and passaged using TrypLE™ Select (Thermo Fisher) to a density of 30–40 K per cm^2^ every 3 or 4 days. For generation of *RYR2* Het KO clones hiPSCs were reverse transfected. Briefly, 500 ng of Puro-pEF1-Cas9-U6-gRNA plasmid, 20 ng of a CMV-GFP plasmid diluted in Opti-MEM™ (Thermo Fisher) and 0.5 µl of PLUS™ reagent (Thermo Fisher), were mixed and added to an equal volume of Opti-MEM and 2.5 µl of Lipofectamine™ LTX reagent (Thermo Fisher). The 500 µl mix was added to the wells of a 12 well plate and incubated for 30 min followed by the addition of 250,000 hiPSCs in 1.5 ml of DEF-CS media containing GF-1,2,3 supplements. Media was changed 24 h post-transfection. At 48 h post-transfection cells were single cell sorted based on GFP expression into 384 well plates using the BD FACSAria™ III (BD Biosciences). Single cell clones were expanded and genomic DNA was extracted using the Purgene Core kit A (Qiagen). PCR was performed using Phusion high fidelity Taq Polymerase (Thermo Fisher). Fragment analysis of PCR products of the clones was performed using 5,200 Fragment Analyzer (Agilent). Next generation sequencing was used to analyse the sequence and mutation integrity through amplicon sequencing using Illumina NextSeq 500 (Illumina). The Wellcome Sanger Institute Genome Editing (wge.stemcell.sanger.ac.uk) and AstraZeneca's CRISPR3 tool (26) were utilized to determine potential off-targets with focus on regulatory sequences where gRNAs had full PAM site complementarity and/or fewer than 5 mismatches with the target. The top 4 putative sites that met these criteria were PCR amplified using Phusion high fidelity Taq Polymerase (Thermo Fisher), followed by electrophoresis and extracted using a gel extraction kit (Qiagen). PCR amplified products were sequenced by Sanger sequencing (GATC Biotech).

### hiPSC characterization

*RYR2* Het KO hiPSCs were analyzed for their pluripotency using the FACS pluripotency marker staining BD stemflow human and mouse pluripotent stem cell analysis kit (BD Biosciences) according to the manufacturer's instructions. In addition, cells were passaged and grown in mTeSR®1 (STEMCELL Technologies) medium in 96 well imaging plates (Perkin Elmer) to obtain colony morphology for immunocytochemistry analysis of pluripotency markers utilizing the human embryonic stem cell marker panel kit (ab109884 AbCam). After fixation in 4% paraformaldehyde (Thermo Fisher), hiPSCs were washed 3 times with DPBS. To permeabilize and block cells, DPBS containing 0.1% Triton-X, 5% FBS was added at room temperature for 1 h. Primary antibodies (OCT4 ab19857 1:1,000, TRA-1-60 ab16288 1:500, SOX2 ab97959 1:1,000, SSEA4 ab16287 1:100) were diluted in DPBS with 5% FBS added to the wells and incubated at 4°C overnight. After DPBS washes, secondary antibodies [AlexaFluor 488 goat anti-rabbit or mouse and AlexaFluor 568 goat anti-rabbit or mouse IgG (H + L)] (Thermo Fisher) were diluted in DPBS containing 5% FBS and Hoechst 1:5,000 and incubated in the dark for 1 h, followed by DPBS washes. Cells were imaged on a Cell Voyager 7,000 (Yokogawa Inc.) using a 20× objective (Olympus UPLSAPO 0.75 NA, 0.6 mm WD), with a 2 × 2 bin. Hoechst stain was imaged using a 405 nm excitation laser (405 ± 5 nm, 100 mw, Coherent) and an Andor Neo sCMOS camera with 445/45 nm band pass emission filter. Alexa Fluor 488 was imaged using a 488 nm excitation laser and a 525/50 band pass filter. Alexa Flour 568 was imaged using a 561 nm excitation laser and a 600/37 band pass emission filter. Images were captured over a 20 µm range in the z-axis, and Z-stack images were output as maximal projection images. Maximum projection images were imported in the Columbus Platform (v2.9.1, Perkin Elmer Inc.). For karyotyping, hiPSCs were grown to 75% confluency in log phase of growth, prepared and shipped to CELL guidance systems for karyotype analyses according to their instructions.

### Cardiomyocyte differentiation

To induce differentiation a previously published protocol was used (27). Briefly, hiPSCs were harvested using TrypLE Select and seeded into 6 well plates pre-coated with Geltrex (Thermo Fisher) at a density of 2.9 × 10^5^ cells/cm^2^ in DEF-CS supplemented medium for 2 h. Media was then changed to basal differentiation media consisting of RPMI 1640 GlutaMAX™ medium (Gibco 61870010), B-27® minus vitamin A (Gibco 12587) and 50 µg/ml ascorbic acid (Sigma-Aldrich A8960) further supplemented with 10 µm CHIR99021 (Tocris Bioscience 4,423) and 80 ng/ml Activin A (R&D systems, 338-AC). At 24 h and 96 h following the initiation of differentiation the media was changed to basal differentiation media supplemented with 5 µm IWR-1 (Sigma-Aldrich, I0161). 120 h post-differentiation induction the media was changed to basal differentiation media only for the remainder of the culture time of 25–30 days, with medium changes every 48 h.

### Western blotting and immunocytochemistry of cardiomyocytes

Cells were differentiated for 25 days and detached using the multi tissue dissociation kit 3 (Miltenyi Biotec) according to the manufacturer's instructions. Cells were pelleted and lysed in ice cold RIPA buffer supplemented with protease and phosphatase inhibitors (Roche) for 30 min with agitation at 4°C. Lysates were centrifuged to remove insoluble fraction. 25 µg of protein lysate was separated on NuPAGE Tris-Acetate 3%–8% midi protein gels (Invitrogen). Proteins were transferred to an Invitrolon™ PVDF membrane (Invitrogen, LC2005) in the Mini Trans-Blot Cell (Bio-Rad) using a Tris-Glycine transfer buffer containing 20% methanol at 80 V. Membranes were blocked in Tris-Buffered Saline with 0.01% Tween 20 (TBS-T) and 5% skim milk powder (Sigma-Aldrich). To detect antigens the membranes were incubated overnight at 4°C with agitation in TBS-T with 5% milk containing diluted primary antibodies (RYR2 R128 clone C3-33 1:1,000 Sigma; ATP2A2 (SERCA2a) ab150435 1:1,000 Abcam; TNNT2 (cardiac troponin T) ab45932 1:1,000 Abcam; Vinculin V19131 1:5,000 Sigma; ACTN2 (sarcomeric alpha-actinin) A7732 1:1,000 Sigma; PLN (Phospholamban) MA3-922 1:100 Thermo Fisher). Membranes were washed 3 times in TBS-T and incubated with secondary antibodies diluted to 1:15,000 (IR dye 800 CW Goat anti-rabbit 925-32211 and IR Dye 800CW Goat anti-mouse 925-32210; LI-COR Biosciences) in TBS-T 10% milk. Membranes were washed and imaged using the LI-COR Odyssey CLx. For immunocytochemistry dissociated cardiomyocytes were plated overnight in 96 well PhenoPlate imaging plates (Perkin Elmer). The following day immunocytochemistry staining was performed following the protocol as described for the hiPSCs with the following primary antibodies (TNNT2 (cardiac troponin T) ab45932 1:500 Abcam; ACTN2 (sarcomeric alpha actinin) A7732 1:500 Sigma; ATP2A2 (SERCA2a) ab150435 1:500 Abcam; PLN (Phospholamban) MA3-922 1:100 Thermo Fisher).

### Flow cytometry of cardiomyocytes

For quantification of TNNT2 (cardiac troponin T), dissociated cardiomyocytes were pelleted and washed twice with 1× perm wash buffer (BD Biosciences), followed by incubation in perm wash buffer for 15 min at room temperature to permeabilize the cells. Cells were centrifuged and resuspended in REA400 cTnT APC labelled (Miltenyi Biotec) diluted 1:100 in perm wash buffer, or REA-APC isotype control followed by incubation in the dark at room temperature for 45 min. Cardiomyocytes were washed twice with perm wash buffer and acquired on a BD Fortessa™ analyzer and analyzed with FlowJo™v10 software (BD Biosciences).

### GSH/GSSG ratio as a measure of oxidative stress

*RYR2* Het KO or WT cardiomyocytes that were differentiated for 25 days were dissociated and plated into 96 well plates coated with fibronectin (Roche) for a further 7 days. Cardiomyocytes were treated with 20 µM of menadione or 0.1% DMSO v/v for 1.5 h. Media was removed and cells were washed 2 times with DPBS. The GSH/GSSG-Glo™ Assay was followed according to the manufacturer's instructions (Promega) and cells were lysed with total or oxidized glutathione reagent.

### Cardiomyocyte functional pharmacological response

WT and *RYR2* Het KO cardiomyocytes were differentiated for 25 days prior to plating at 100k cells per well of an xCELLigence® RTCA CardioECR (Agilent Technologies) plate coated with Geltrex. Cardiomyocyte maintenance media was changed every 48 h and cells were used after a further 10 days of culture. On the day of compound addition verapamil or nifedipine (Sigma-Aldrich) were dissolved in DMSO to give stock concentrations. Semi-log 1× drug concentrations in maintenance media with 0.1% final DMSO v/v concentration were made and put into a V-bottom polypropylene dosing plate that was sealed with a breathable seal and pre-equilibrated in a cell culture incubator for 20–30 min. The xCELLigence® RTCA CardioECR instrument (Agilent Technologies) was used for impedance recording with an interval setting of 2 min, block duration of 60 s and cardio speed 12 ms. Where cells were paced, stimulus settings were pulse type, Amp (mV) 1,500.00, length 20.00 ms, and frequency 1 Hz. Maintenance media was re-freshed and 3–4 h later a baseline recording was made. For compound addition the plate was removed from the instrument and a cumulative concentration response protocol was performed where 90% of the media was removed and replaced with either 0.1% DMSO v/v control or the lowest concentration of drug (1 nM). The plate was placed back onto the instrument and after 5 min equilibration period the recording was initiated. This was repeated in a cumulative manner with addition of increasing drug concentrations. Recordings were analysed using the RTCA CardioECR data analysis software and beat rate per minute and beat amplitude were calculated 10 min after each sequential compound addition.

### Cardiomyocyte transfection with siRNA and functional analysis

WT and *RYR2* Het KO differentiated cardiomyocytes were dissociated and replated into Nanion CardioExcyte 96 NSP 2.0 mm (Nanion Technologies) plates that were coated with fibronectin at 60,000 per well, with media change 48 h later. 4 days after plating cardiomyocytes were transfected with siRNAs using ON-TARGETplus Human CSRP3, G6PD or ON-TARGETplus non-targeting control siRNA (Dharmacon) following the manufacturer's instructions using 100 nM final concentration of siRNA and 0.5 µl of DharmaFECT1 reagent (Dharmacon) per 96 well. 72 h after transfection recordings of spontaneously beating cells were made using the CardioExcyte 96 instrument (Nanion Technologies) using the impedance mode, a repetition interval of 5 min and sweep duration of 30 s. At 96 h post-transfection after a baseline recording, the cardiomyocytes were treated with 30 nM, 100 nM of verapamil or 0.1% DMSO v/v. Recordings were analyzed 30 min post compound addition for beat rate and beat amplitude using the Data Control 96 version 1.8.0 build 27 (Nanion Technologies). After conclusion of experiments samples were harvested for western blot of the CSRP3 or G6PD protein expression following the protocol described earlier [primary antibodies used were G6PD (D5D2) #12263, 1:1,000, Cell Signaling Technology, CSRP3 ab173301 1:1,000, Abcam].

### Calcium handling

Cardiomyocytes were plated on to glass coverslips coated in Geltrex and cultured for a further 10 days. Cells were loaded with Fluo4 (1 µM final concentration) in Tyrode's buffer (137 mM NaCl, 5.0 mM KCl, 1.0 mM MgCl2, 1.0 mM CaCl2, 10 mM HEPES, and 10 mM glucose, pH 7.4) for 30 min at 37°C. Cells were washed once in Tyrode's buffer and imaged at room temperature using a Zeiss LSM 800 Confocal microscope. Fluo4 fluorescence was stimulated by 488 nm laser, and emission captured between 510 and 550 nm with an image taken every 250 ms. Calcium transients were analyzed using ImageJ.

### Quantitative proteomics analysis by LC-MS

Cardiomyocytes that had been differentiated for 30 days were harvested from 6 well plates. Seven cell pellet aliquots (3× *RYR2* Het KO 2 and 4× WT control) of ca. 2 × 10^6^ cardiomyocytes each were lysed and proteome-extracted in 0.5% SDS, 0.1 M Tris*HCl pH 8.5, 0.1 M DTT at 90°C for 5 min followed by centrifugation at 20,000 g for 30 min. A fraction of cleared supernatants were transferred to spin filters (Microcon, Millipore) and processed according to the FASP protocol (28). Briefly, cells were washed multiple times with 8 M urea, 0.1 M Tris*HCl pH 8.5, alkylated with 0.05 M iodoacetamide in 8 M urea in 0.1 M Tris*HCl, pH 8.5 and washed again, followed by dilution with 1M urea, 0.05 M ammonium bicarbonate to <1.5 M urea. Trypsin was added at a protein-to-trypsin (m/m) ratio of 50:1 and digested for 18 h at 30°C. Tryptic peptides were harvested by centrifugation and desalted using C18 Stage Tips (29).

LC-MS/MS analyses were performed using an Orbitrap Fusion Lumos Tribrid mass spectrometer (Thermo Fisher Scientific, Germany) connected to an Ultimate 3,000 RSLC nano system (Dionex, Thermo Fisher Scientific, Germany). After resuspension in 1.6% acetonitrile 0.1% formic acid, samples were separated on an EASY-Spray column of 50 cm length (Thermo Scientific) running at 300 nl/min. Peptides were eluted using the following gradient of water with 0.1% formic acid (solvent A) and 80% acetonitrile with 0.1% formic acid (solvent B): 0–142.5 min 2%–45% B, 142.5–147.5 min 45%–50% B, 147.5–154 min 50%–90% B, 154–159 min 90% B, 159–160 min 90%–2% B, 160–185 min 2% B.

The mass spectrometer was operated in data-dependent mode using the Top-speed setting with 2 s cycle time. The MS1-scan resolution in the Orbitrap was set to 120k over 375–1,500 m/z, with maximum injection time set to “Automatic” and a normalized automatic gain control (AGC) target to 250%. Precursor ions passing a 5e3 intensity cut-off and fulfilling monoisotopic precursors filter with z = 2–6+ were selected for fragmentation by higher-energy collision-induced dissociation (HCD) with 30% normalized collision energy (NCE) followed by detection in the Ion trap operated in rapid scan mode at a maximum injection time setting of “dynamic” and the normalized AGC target set to 200%. Dynamic exclusion was enabled upon a single observation for a precursor ±5 ppm including isotopes for 40 s. LC-MS data were processed using MaxQuant 1.6.10.43 with default settings (protein FDR 0.01, min. peptide length 7, revert decoy mode and razor peptides used for quantification) against the human Uniprot UP000005640 database (reviewed proteins only).

### Bioinformatics analysis of LC-MS data

Label Free Quantification (LFQ) values from the proteinGroups.txt file from MaxQuant were used for differential protein expression analysis. Potential contaminants, reverse hits and proteins only identified by site were removed. Furthermore, only proteins quantified using more than one peptide were used for downstream analytics. The LFQ values were log2-normalised and centered by subtracting the sample median values. Missing values were imputed only for control samples (due to there being enough samples to perform this operation). The approach to imputation was as follows: for proteins missing at most in one control sample, a mean of the three remaining samples was used for imputation. If a value was missing in more than one sample, no imputation was performed. This control-imputed data matrix, containing 4,907 protein groups, was used for downstream analysis. One heterozygote sample was removed from differential protein expression analysis due to being a suspected outlier using Pearson correlation analysis and PCA. Limma 3.54.2 R package (30) was used for performing differential expression analysis (2× heterozygote samples vs. 4× controls), using the eBayes procedure with trend parameter set to “True”. Overrepresentation analysis was carried out using KEGG pathways and Qiagen Ingenuity Pathway Analysis (IPA), and the complete list of analyzed proteins as the background set. Network analysis of the pentose phosphate pathway was performed using IPA.

### Statistical methods

Statistical methods are stated within each figure legend. Briefly, an unpaired two-tailed *t*-test was used when comparing mean functional responses between two different genotypes (for example, CTR siRNA vs. either CSRP3 or G6PD siRNA treated cells). A one-way Anova statisitcs test with Sidak's multiple comparisons was used to compare specific protein expression, or mean beat rate and beat amplitude effects in WT cells compared to either Het KO 1 or Het KO 2 cells. A two-way Anova statistics test with Sidak's multiple comparisons was used when comparing mean functional effects of matching compound treatments across different genotypes or CTR vs. gene-specific siRNA treated cells.

## Results

### hiPSC CRISPR/Cas9 gene editing and characterization

**Figure 1:**
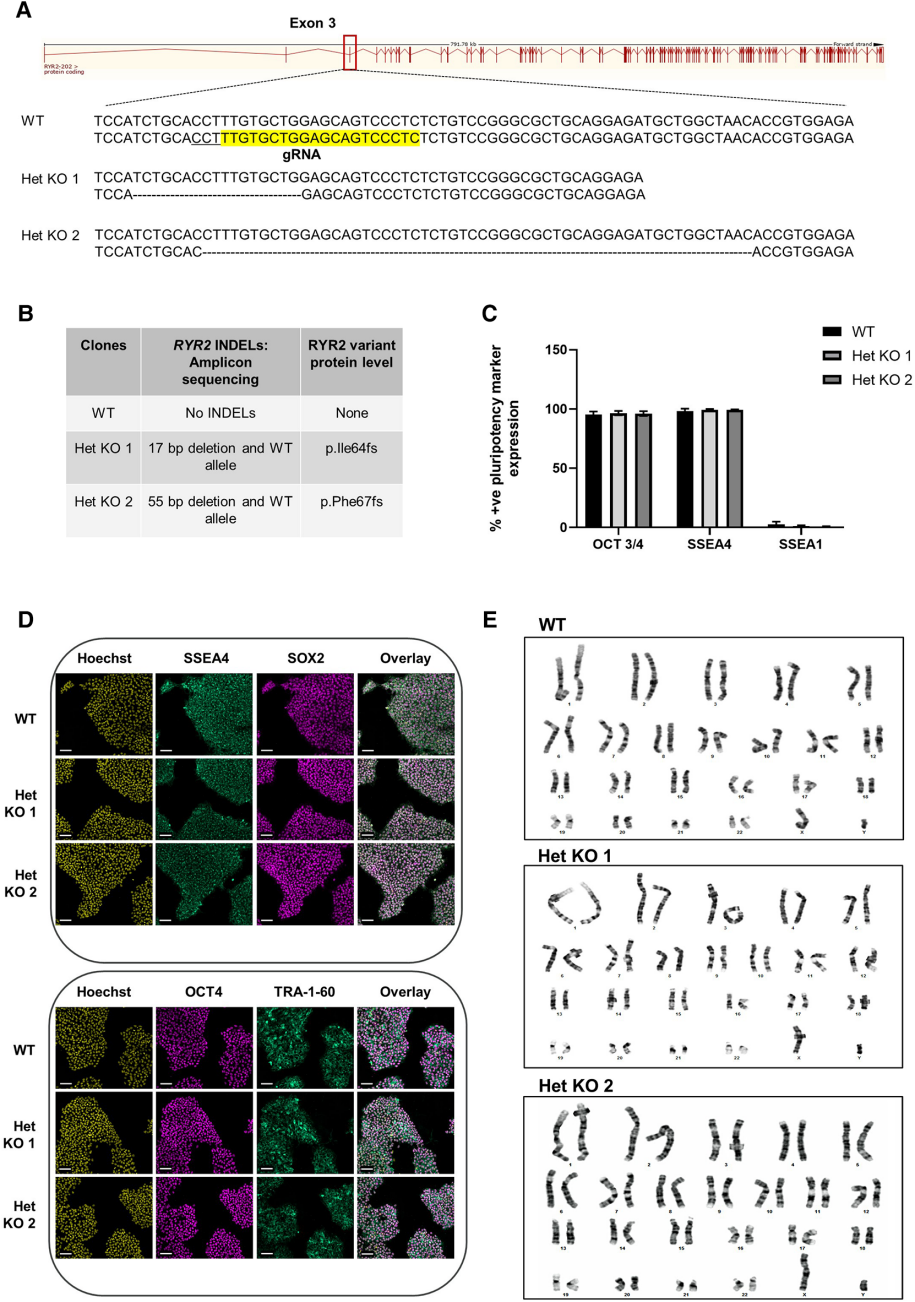
CRISPR/Cas9 generation of heterozygous knockout *RYR2* pluripotent hiPSCs. (**A**) Schematic of guide RNA (sequence highlighted in yellow and PAM site underlined) targeting one allele of exon 3 of the *RYR2* gene. Depicted are the resulting heterozygous INDELs characterized in 2 heterozygous knockout clones (Het KO 1, Het KO 2). (**B**) Table shows the INDELS characterized by targeted-amplicon sequencing and the resulting heterozygous variant at the protein level. (**C**) Average percent positive pluripotency marker expression for OCT 3/4, SSEA4 and differentiation marker SSEA1 in hiPSCs as measured by flow cytometry. Mean ± SEM, WT (*n* = 4), Het KO 1 (*n* = 3), Het KO 2 (*n* = 3) (**D**) Representative immunocytochemistry images of pluripotent marker (SSEA4, SOX2, OCT4, TRA-1-60) staining of WT and 2 heterozygous *RYR2* knockout hiPSCs (*n* = 4). Hoechst indicates nuclei, overlay indicates Hoechst, SSEA4 and SOX2 or Hoechst, OCT4 and TRA-1-60 combinations. Scale bar represents 100 µm. (**E**) Representative images of G-banding karyotype of WT parental, 2 heterozygous *RYR2* knockout hiPSCs (*n* = 20 cells each genotype).

To develop a genetically engineered cardiovascular model with reduced RYR2 expression, heterozygous *RYR2* knockout clones were generated using CRISPR/Cas9 methodology. hiPSCs were co-transfected with a CRISPR/Cas9 gRNA-containing plasmid containing a gRNA targeting exon 3 (Figure 1A) and a GFP plasmid. GFP positive cells were single-cell FACS sorted and expanded followed by Cel 1 PCR assay of genomic DNA to identify clones with INDELs, that were further analyzed using a targeted amplicon-sequencing approach to determine the genetic sequence of the targeted *RYR2* locus at exon 3. WT cells refer to isogenic control cells. Three heterozygous *RYR2* knockout (Het KO) clones were obtained, two containing several base pair deletions (Figures 1A,B) and one with a single base pair insertion (Supplementary Figures S1A,B) all resulting in an out of frame mutation on 1 allele. Pluripotency of the hiPSCs was measured to ensure that this was unaffected following the gene-editing and selection procedures. WT and *RYR2* Het KO clones maintained expression of key pluripotent markers (SSEA4, SOX2, OCT4, TRA-1-60) quantified by flow cytometry (Figure 1C) and visualized by confocal microscopy (Figure 1D, Supplementary Figure S1C). In addition, a quality control screening for genetic integrity and identification of chromosomal abnormalities was performed by G-band karyotyping. Both WT and *RYR2* Het KO clones maintained a normal 46XY karyotype indicating maintenance of genomic integrity during gene-editing and clone selection (Figure 1E and Supplementary Figure S1D). Potential phenotypes observed in the *RYR2* Het KO clones could be due to off-target events rather than the *RYR2* Het KO *per se*, so this was determined across all 3 clones utilizing the Wellcome Sanger Institute Genome Editing (wge.stemcell.sanger.ac.uk) and AstraZeneca's CRISPR3 tool (26) with focus on regulatory sequences where gRNAs had full PAM site complementarity and/or fewer than 5 mismatches with the target. The top 4 putative sites that met these criteria were PCR amplified and analyzed by Sanger sequencing and found not to be affected (Supplementary Figure S2).

### Cardiomyocyte differentiation of RYR2 hiPSCs

**Figure 2:**
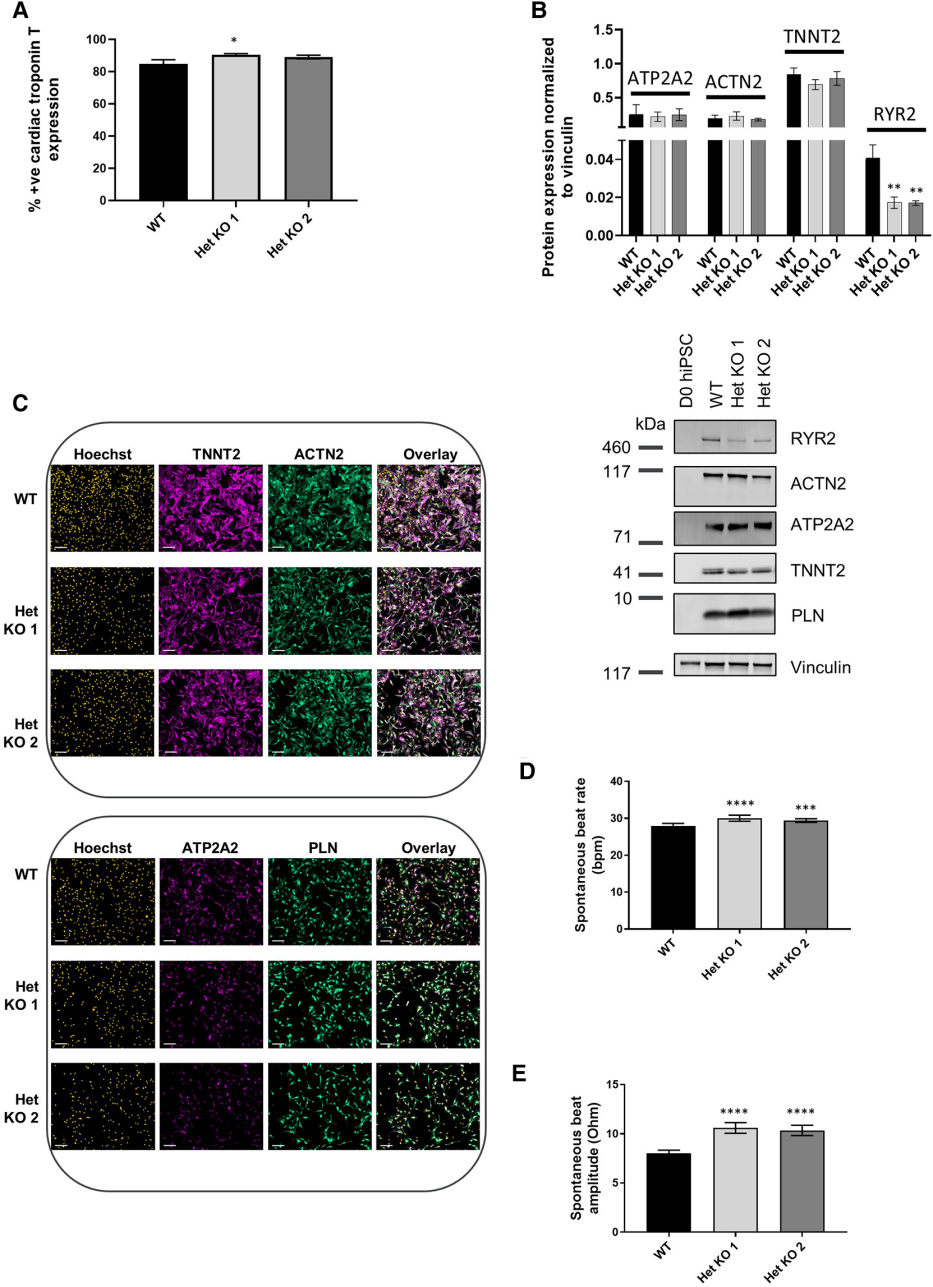
*RYR2* hiPSCs differentiate to cardiomyocytes. (**A**) Average percent positive cells for cardiac troponin T expression measured by flow cytometric analysis at day 25 of differentiation in WT, Het KO 1 and Het KO 2 hiPSC-derived cardiomyocytes (hiPSC)-CMs. Mean ± SEM, WT (*n* = 6), Het KO 1 (*n* = 10), Het KO 2 (*n* = 8) from at least 3 differentiations. (**B**) Top: quantification of western blots from WT, Het KO 1, Het KO 2 hiPSC-CMs at day 25 of differentiation. Mean ± SD (*n* = 3). Bottom: representative western blot of RYR2, ATP2A2, ACTN2, TNNT2, PLN protein expression and vinculin as a loading control. (**C**) Representative immunocytochemistry images of TNNT2, ACTN2, ATP2A2 and PLN protein expression in WT and Het KO 1, Het KO 2 hiPSC-CMs. Hoechst indicates nuclei, overlay indicates Hoechst, TNNT2, ACTN2 or Hoechst, ATP2A2, PLN combinations. Scale bar represents 100 µm (*n* = 4). (**D**) Impedance-based measurements of spontaneous beat rate of WT, Het KO 1 and Het KO 2 hiPSC-CMs measured after 25 days of differentiation. Mean ± SEM (*n* = 8 per cell type). (**E**) Impedance-based measurements of beat amplitude of WT, Het KO 1 and Het KO 2 hiPSC-CMs measured after 25 days of differentiation. Mean ± SEM (*n* = 8 per cell type). Statistics in figures are performed by one-way ANOVA with Sidak's multiple comparisons test; *p-value <0.05, **p-value <0.01, ***p-value <0.001, ****p-value <0.0001.

**Figure 3:**
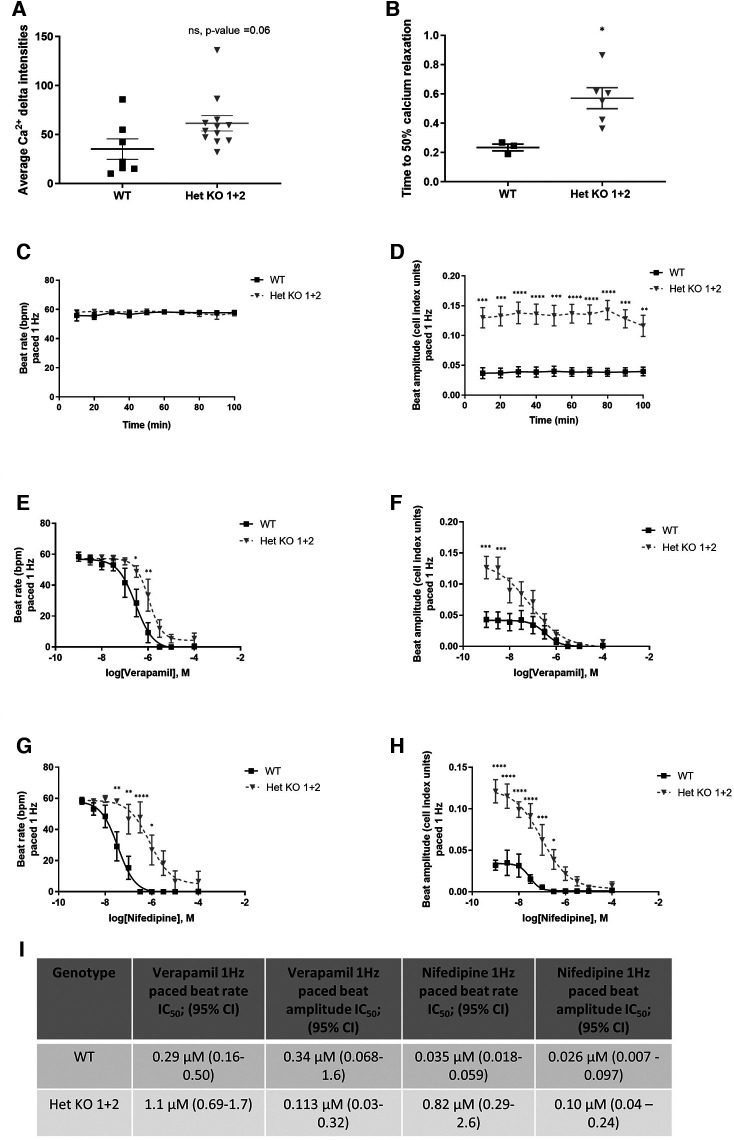
*RYR2* hiPSC-CMs calcium handling and differential pharmacological response. (**A**) Average calcium peak intensities and (**B**) calcium decay half life kinetics for WT and Het KO hiPSC-CMs. Confocal line-scanning microscopy was performed and average Ca^2+^ delta intensities, Mean ± SEM (WT *n* = 7, Het KO *n* = 12), and time to 50% calcium relaxation, Mean ± SEM (WT *n* = 3, Het KO *n* = 6) from at least 3 differentiations were measured. Statistics used are unpaired two-tailed t-tests; *p-value <0.05. (**C**) Timecourse assessment of beat rate and (**D**) beat amplitude in WT and *RYR2* Het KO hiPSC-CMs paced at 1 Hz and treated with 0.1% DMSO (v/v). Mean ± SEM (WT *n* = 6, Het KO *n* = 12) from at least 3 differentiations. (**E–H**) WT and *RYR2* Het KO cells were treated in a cumulative concentration response and paced at 1 Hz. Beat rate and beat amplitude measurements were analyzed 10 min after each cumulative concentration addition of (**E,F**) verapamil, Mean ± SEM (WT *n* = 6, Het KO *n* = 7) or (**G,H**) nifedipine. Mean ± SEM (WT *n* = 6, Het KO *n* = 6) from at least 3 differentiations. (**I**) Table shows calculated IC_50_ values and 95% confidence intervals for beat rate and beat amplitude of verapamil and nifedipine treated cells. Statistics in figures are performed by two-way ANOVA with Sidak's multiple comparisons test; *p-value <0.05, **p-value <0.01, ***p-value <0.001, ****p-value <0.0001.

*RYR2* Het KO 1 and Het KO 2 as well as WT hiPSCs were differentiated to cardiomyocytes for 25 days and structurally characterized prior to investigation of functional parameters including contractility and calcium handling properties. Similar cardiomyocyte differentiation efficiencies were obtained between WT and *RYR2* Het KO clones with average percentage values of 85%–91% TNNT2 (cardiac troponin T) positive cells from independent differentiations. Flow cytometry measurements of TNNT2% were performed prior to functional analyses to ensure similar expression levels across clones being compared (Figure 2A). RYR2, ATP2A2, TNNT2 and PLN were measured by western blot and immunocytochemistry. *RYR2* Het KO clones showed a decrease in RYR2 expression (49%–61%) compared to WT cells. Other cardiomyocyte specific markers including TNNT2, ACTN2, ATP2A2, and PLN were expressed with no significant differences observed between the two genotypes (Figures 2B,C). Functional analyses of beat rate and beat amplitude were performed using impedance-based measurements. *RYR2* Het KO 1 and KO 2 clones (30.0 bpm ± 0.82, 29.4 bpm ± 0.5) displayed similar spontaneous beat rates to WT (27.9 bpm ± 0.66) and a 1.3-fold increased amplitude with respect to WT cells (Figures 2D,E). As *RYR2* Het KO 1 and 2 clones showed similar RYR2 reduction levels, spontaneous beat rates and beat amplitude measurements, further functional analyses were carried out from combined and averaged readings from RYR2 Het KO 1 and 2 clones. Confocal line-scanning microscopy of calcium signaling in the cells showed a trend of increased calcium peak intensities for *RYR2* Het KO cells vs. WT cells, but there was a significant delayed time to relaxation, suggesting a larger release of calcium from the sarcoplasmic reticulum that is not being removed as quickly after each beat as it is for WT cells (Figures 3A,B, Supplementary Figure S3). This is reminiscent of the calcium handling dysfunction in mice that are haplo-insufficient for *Ryr2* (21). The calcium handling pathway in *RYR2* Het KO vs. WT cardiomyocytes was interrogated by pharmacological inhibition of the L-type calcium channel. hiPSCs that were differentiated to cardiomyocytes (hiPSC-CMs) for 25 days were plated in impedance-based plates for 10 days followed by treatment with verapamil or nifedipine in a cumulative dose-response manner. Beat rate and beat amplitude measurements were performed on cells paced at 1 Hz and time-matched vehicle (0.1% DMSO v/v) controls were included for comparison. Over a time course assessment, *RYR2* Het KO cells showed an equivalent beat rate to WT cells treated with 0.1% DMSO v/v paced at 1 Hz (Figure 3C), but the beat amplitude was increased (Figure 3D), in alignment to the trend of an increased calcium peak intensity, along with the slower calcium uptake (Figure 3B). All hiPSC-CMs showed a concentration-response with decreased beat rate and beat amplitude in response to verapamil (Figures 3E,F) and nifedipine (Figures 3G,H) treatment. The beat rate response to verapamil of WT cells [IC_50_ 0.29 µM (0.16–0.50)] was left-shifted compared to *RYR2* Het KO cells [IC_50_ 1.1 µM (0.69–1.7)]. This trend was also shown for nifedipine; WT cells had an IC_50_ of 0.035 µM (0.018–0.059) and *RYR2* Het KO cells IC_50_ 0.82 µM (0.29–2.6) (Figure 3I). To confirm the significant increase in beat amplitude upon pacing, the *RYR2* Het KO 3 clone was also differentiated to cardiomyocytes for 25 days and similarly showed reduced expression of RYR2 protein, similar expression of TNNT2 and additional cardiac markers ACTN2, ATP2A2, PLN (Supplementary Figures S1E–G). Upon pacing at 1 Hz *RYR2* Het KO 3 cardiomyocytes confirmed the equivalent beat rate to WT cells and an increased beat ampitude (2.4-fold increase compared to WT cells (Supplementary Figures S1H,I). Altogether, these results indicate that the *RYR2* Het KO cells exhibit a trend of increased cytosolic calcium with slowed re-uptake kinetics and are less sensitive to L-type calcium channel inhibition.

### Characterization of cardiomyocytes via global proteomics

**Figure 4:**
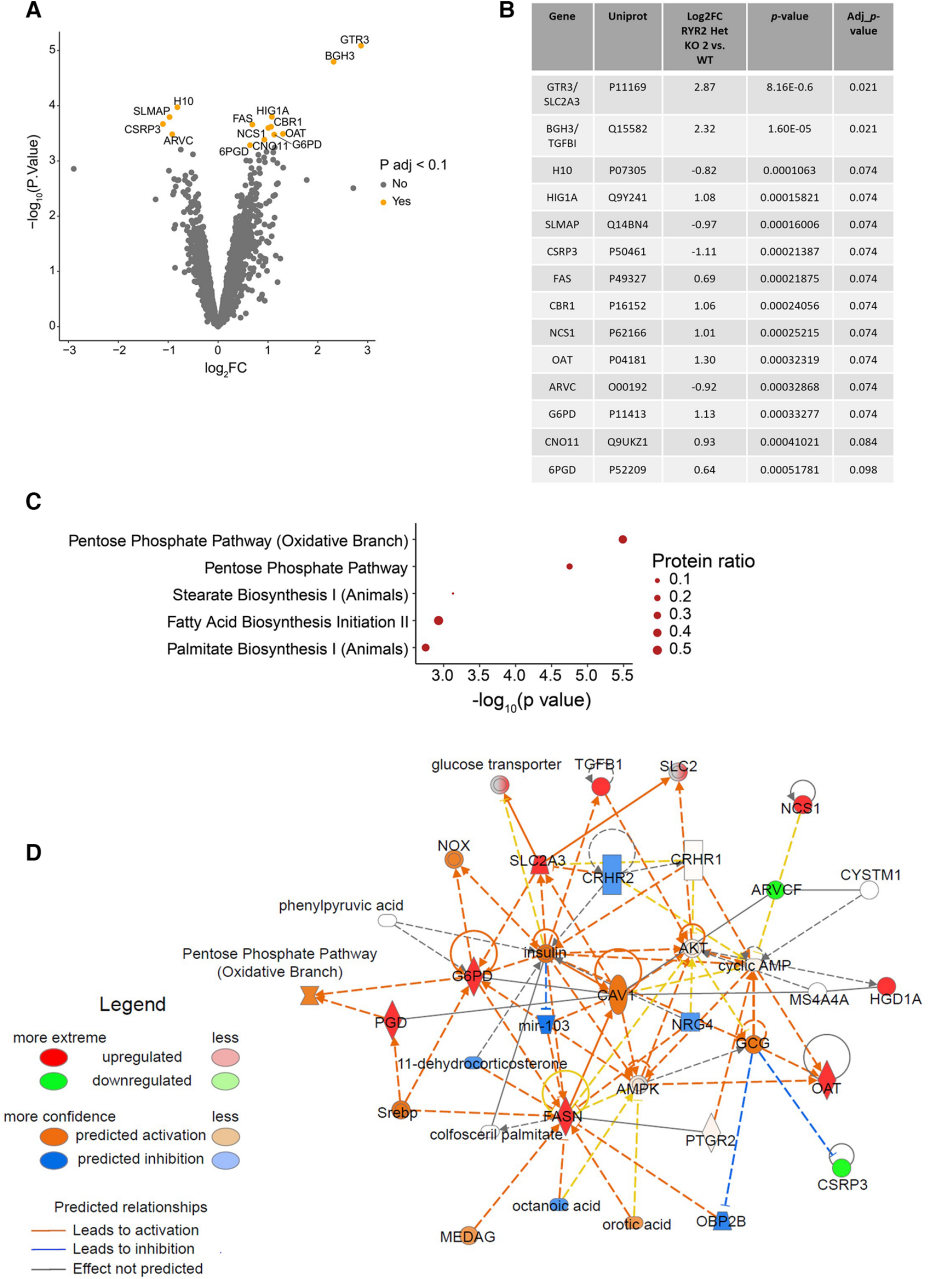
*RYR2* Het KO 2 hiPSC-CMs show an upregulation of the pentose phosphate pathway. (**A**) Volcano plot showing proteins with altered expression in *RYR2* hiPSC-CMs compared to WT. Proteins with a Benjamini-Hochberg adjusted *p*-value <0.1 are labelled and highlighted in orange. (**B**) Table summarising changes in protein expression in *RYR2* Het KO 2 hiPSC-CMs compared to WT, for the 14 proteins with an adjusted *p*-value <0.1. (**C**) KEGG overrepresentaion analysis of proteins with adjusted *p*-value <0.1, in the context of all proteins analysed. Top 5 enriched pathways are shown. Protein ratio shows proportion of proteins with adjusted *p*-value <0.1, compared to all proteins included in analysis for each pathway. (**D**) Overview of changes in expression of members of the pentose phosphate pathway using IPA. Upregulated proteins, including SCL2A3, OAT and G6PD are highlighted in red, and downregulated proteins including CSRP3 and SLMAP are highlighted in green, with shading indicating magnitude of expression change. Confidence in relationships between pathway members is indicated by blue/orange lines (inhibition or activation respectively).

To gain mechanistic understanding of the functional differences in *RYR2* Het KO cells and their differential pharmacological response an unbiased label-free global proteomics approach was taken. To manage the workflow focus was placed on WT and characterization of one of the heterozygous KO clones. To this end, WT and *RYR2* Het KO 2 hiPSCs were differentiated into cardiomyocytes for 30 days, followed by lysate collection. Label-free quantitative mass spectrometry was performed and identified 4,907 unique proteins. One *RYR2* Het KO 2 sample was removed from analysis as it was a suspected outlier using Pearson correlation and pinciple component analysis (Supplementary Figure S4). After standard filtering (Benjamini-Hochberg adjusted *p*-value <0.1) 14 proteins were found to have altered expression in *RYR2* Het KO 2 compared to WT hiPSC-CMs (Figures 4A,B). The adjusted *p*-value <0.1 corresponds to an expected 10% of false positives within the list of 14 proteins. Focus was placed on interpretation of pathway anaysis where evidence of several proteins acting in a common pathway provides confidence to the findings, given the reduced number of *RYR2* Het KO 2 samples. Indeed, KEGG overrepresentation analysis of the 14 proteins showing altered expression, i.e., adjusted *p*-value <0.1, identified the enrichment of the pentose phosphate pathway (adjusted *p*-value* *= 7.16e-03) in *RYR2* Het KO 2 cells (Figure 4C). As shown by the proteins highlighted in red in Figure 4D, a number of the identified, upregulated proteins, such as OAT and G6PD are within this common pathway, while SLC2A3 was identified as a significantly enriched protein. These findings support the view of increased capacity for glucose uptake and shunt to the pentose phosphate pathway in *RYR2* Het KO 2 cells (Figures 4A–D). Outside the pentose phosphate pathway, the other significantly enriched protein upregulated in *RYR2* Het KO 2 cells is TGFBI which is upregulated and correlated to aging in DCM cardiomyocytes (31). Additionally, CSRP3 and SLMAP that were identified as possibly downregulated are known calcium handling modulators and are dysregulated in heart failure (32–34), with downregulation of CSRP3 in a *Ryr2* KO mouse model (22).

### Mechanistic insights into functional differences and pharmacology responses

**Figure 5:**
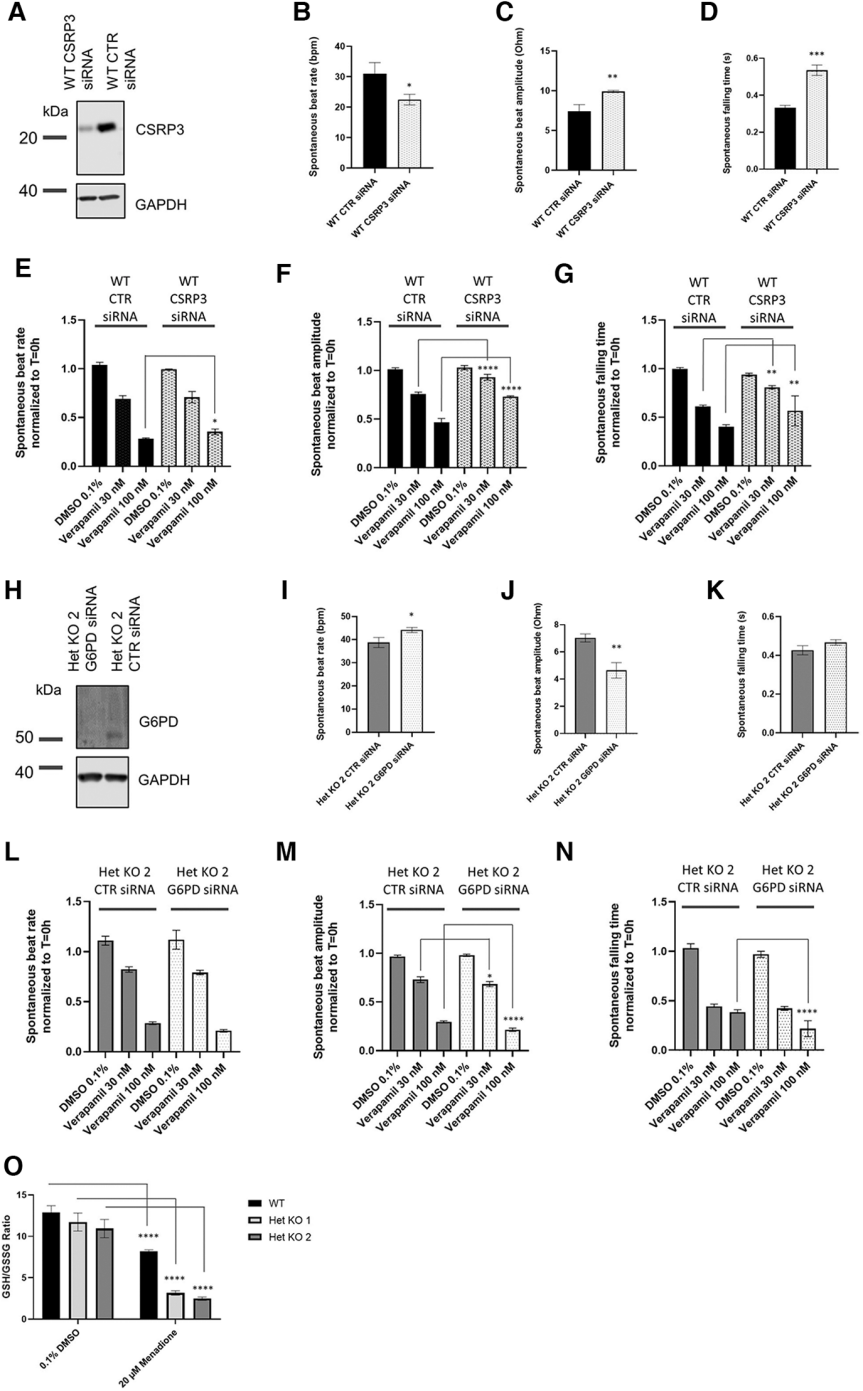
Mechanistic insights from proteomics. (**A**) Representative western blot of lysates from control (CTR) siRNA and CSRP3 siRNA transfected WT hiPSC-CMs collected from 0.1% DMSO v/v treated wells after completion of impedance-based measurements (*n* = 3). (**B**) WT hiPSC-CMs were transfected with CTR or CSRP3 siRNA. 96 h post-transfection spontaneous beat rate, (**C**) beat amplitude and (**D**) falling time were measured (*n* = 3). Statistics used are unpaired two-tailed *t*-tests; *p-value <0.05, **p-value <0.01. (**E**) Beat rate, (**F**) beat amplitude or (**G**) falling time response to 0.1% DMSO v/v treatment or 30 nM or 100 nM verapamil were measured 30 min post-treatment. Data are shown as normalized to T = 0 h that is the baseline prior to 0.1% DMSO v/v or drug treatment. Mean ± SEM (*n* = 4). (**H**) Representative western blot of lysates from CTR and G6PD siRNA transfected Het KO 2 cardiomyoctyes collected from 0.1% DMSO v/v treated wells after completion of impedance-based measurements (*n* = 3). (**I**) Het KO 2 hiPSC-CMs were transfected with CTR or CSRP3 siRNA. 96 h post-transfection spontaneous beat rate, (**J**) beat amplitude and (**K**) falling time were measured (*n* = 3). (**L**) Beat rate, (**M**) beat amplitude or (**N**) falling time response to 0.1% DMSO v/v treatment or 30 nM or 100 nM verapamil 30 min post-treatment. Data are shown as normalized to T = 0 h that is the baseline prior to 0.1% DMSO v/v or drug treatment. Mean ± SEM (*n* = 4). Statistics two-way ANOVA with Sidak's post-test for multiple comparisons comparing CTR siRNA transfected cells to either CSRP3 or G6PD siRNA cells and matching verapamil treatment concentrations. *p-value <0.05, **p-value <0.01, ***p-value <0.001, ****p-value <0.0001. (**K**) GSH/GSSG ratio measured in WT, *RYR2* Het KO 1, Het KO 2 cells differentiated for 25 days, 1.5 h post treatment with 0.1% DMSO v/v or 20 µM Menadione. Mean ± SEM (*n* = 3). Statistics two-way ANOVA with Sidak's post-test for multiple comparisons comparing to 0.1% DMSO v/v control; ****p-value <0.0001.

Targets that were identified from the proteomics approach were followed up with functional interrogation using siRNA and impedance-based analysis of beat rate, beat amplitude and falling time to link to the observed changes in calcium handling in Figure 3B. *RYR2* Het KO cardiomyocytes showed an increased beat amplitude at baseline and a decreased sensitivity to verapamil, an L-type calcium channel blocker. Therefore, it was hypothesized that proteins upregulated in *RYR2* Het KO when knocked down may result in a reduction in beat amplitude and increased verapamil sensitivity, whereas proteins that were downregulated in *RYR2* Het KO cardiomyocytes if reduced in WT cardiomyocytes may increase their beat amplitude and reduce sensitivity to verapamil. In alignment with the proteomics results, recent publications in *Ryr2* KO mice have shown reduced expression of CSRP3 (22) and *CSRP3* KO hESC-CMs exhibit a hypertrophic cardiomyopathy phenotype, with dysfunctional calcium handling (32). Because of the existing literature evidence CSRP3 was chosen and knocked down in WT hiPSC-CMs that were subjected to verapamil treatment. Western blot showed 78% knockdown of protein 96 h post-transfection when responses were measured (Figure 5A). CSRP3 siRNA transfected cells showed a reduced beat rate compared to control (CTR) cells (22.5 bpm ± 1.02 vs. 30.9 bpm ± 2.14) (Figure 5B), an increased beat amplitude compared to CTR cells (9.9 Ohm ± 0.08 vs. 7.4 Ohm ± 0.49) (Figure 5C) and an increased falling time (0.54 s ± 0.03 s vs. 0.33 s ± 0.01) (Figure 5D). In response to verapamil, WT CTR siRNA transfected cells showed a reduction in beat rate (1.5-fold and 3.7-fold with 30 nM and 100 nM of verapamil, respectively). In comparison, WT CSRP3 siRNA transfected cells showed a similar reduction in beat rate with 30 nM verapamil (1.4-fold) and a lesser reduction upon 100 nM verapamil treatment (2.8-fold) (Figure 5E). In WT CTR siRNA transfected cells, beat amplitude was reduced by 1.3-fold and 2.1-fold with 30 nM and 100 nM of verapamil, respectively. Whereas, in CSRP3 siRNA transfected cells beat amplitude was affected to a lesser extent upon verapamil treatment (1.1-fold and 1.4-fold with 30 and 100 nM of verapamil, respectively) (Figure 5F). In WT CTR siRNA transfected cells, falling time was reduced by 1.6-fold and 2.5-fold with 30 and 100 nM of verapamil respectively. Whereas, in CSRP3 siRNA transfected cells the falling time was affected to a lesser extent upon verapamil treatment (1.2-fold and 1.7-fold with 30 and 100 nM of verapamil, respectively) (Figure 5G). These results suggest that a decrease in CSRP3 expression *per se* can modulate the response to L-type calcium channel inhibition, making cells less sensitive to reductions in beat rate, beat amplitude and falling time that may partially explain the pharmacological phenotype of *RYR2* Het KO cells.

Importantly, the proteomics analysis identified an upregulation of the pentose phosphate pathway, that to our knowledge is novel and has not been described before as being affected by RYR2 protein expression levels. The pentose phosphate pathway accounts for approximately 60% of NADPH production in humans, which in turn helps to maintain redox homeostasis of the cell via the regeneration of reduced glutathione. NADPH is utilized to fuel antioxidant systems and decrease reactive oxygen species (ROS). The GLUT3 receptor (SLC2A3) was found to have strong upregulation and we hypothesized that *RYR2* Het KO cells have an increased glucose uptake and are shunting glucose utilisation to the pentose phosphate pathway. Glucose 6-phosphate dehydrogenase (G6PD) controls the flux into the pentose phosphate pathway. Since G6PD was found to be upregulated, it was knocked down in *RYR2* Het KO 2 cells that were subsequently treated with verapamil. Western blotting confirmed 97% knockdown of G6PD protein at 96 h when responses were measured (Figure 5H). At baseline G6PD siRNA transfected cells showed a slight increase in beat rate compared to CTR siRNA transfected cells (44.2 bpm ± 0.94 vs. 38.8 bpm ± 1.3) (Figure 5I), a decreased beat amplitude compared to CTR cells (4.6 Ohm ± 0.33 vs. 7.0 Ohm ± 0.17) (Figure 5J) and no significant change in falling time (0.47 s ± 0.01 vs. 0.43 ± 0.02) (Figure 5K). Upon verapamil treatment *RYR2* Het KO 2 CTR siRNA transfected hiPSC-CMs showed a decreased beat rate (1.3-fold, 3.9-fold with 30 nM and 100 nM verapamil, respectively). *RYR2* Het KO 2 G6PD siRNA transfected cells showed a greater effect on beat rate reduction upon 100 nM verapamil treatment (1.4-fold, 5.4-fold with 30 nM and 100 nM verapamil, respectively) (Figure 5L). Compared to *RYR2* Het KO 2 CTR siRNA transfected cells (1.3-fold, 3.2-fold reductions in beat amplitude with 30 nM and 100 nM verapamil, respectively), *RYR2* Het KO 2 G6PD siRNA transfected cells showed a greater response to 100 nM of verapamil treatment (1.4-fold, 4.5-fold reductions in beat amplitude with 30 nM and 100 nM verapamil, respectively) (Figure 5M). Compared to *RYR2* Het KO 2 CTR siRNA transfected cells (2.3-fold, 2.7-fold reductions in falling time with 30 nM and 100 nM verapamil, respectively), *RYR2* Het KO 2 G6PD siRNA transfected cells showed a greater response to 100 nM of verapamil treatment (2.3-fold, 4.5-fold reductions in falling time with 30 nM and 100 nM verapamil, respectively) (Figure 5N). These results suggest an increased sensitivity to L-type calcium channel inhibition when the pentose phosphate pathway is limited.

The redox status of the hiPSC-CMs may be altered, therefore we examined the GSH/GSSG ratio within WT and *RYR2* Het KO 1 and 2 cardiomyocytes in the presence and absence of menadione. Intracellular levels of glutathione in reduced form (GSH) and oxidized form (GSSG) as well as the GSH/GSSG ratio are critical for redox homeostasis of the cell. A small portion of glutathione in a cell is in the oxidized state (GSSG) and is an indicator of oxidative stress in cells. Menadione increases glutathione oxidation (GSSG), leading to increased ROS levels. Whilst *RYR2* Het KO 1 and 2 cardiomyocytes displayed slightly reduced GSH/GSSG ratios compared to WT, although not significant, they showed a 4-fold reduction in response to menadione treatment, whereas WT cells showed a 1.6-fold reduction (Figure 5O).

Altogether our results show that hiPSC-CMs that are heterozygous for RYR2 expression show calcium handling dysfunction, altered glucose metabolism, differential pharmacological responses and increased sensitivity to redox alterations, and serve as a genetically engineered model for mimicking some aspects of heart failure phenotypes.

## Discussion

In this study an *in vitro* genetically engineered model for RYR2 protein reduction was developed using CRISPR/Cas9 to edit the *RYR2* locus of hiPSCs to make a heterozygous knockout. *RYR2* Het KO hiPSCs efficiently differentiated to cardiomyocytes expressing ∼50% of the RYR2 protein compared to WT suggesting that RYR2 *per se* is dispensable for cardiomyocyte differentiation, in line with a previous report showing that complete absence of RYR2 is dispensable for ES cell differentiation to cardiomyocytes (35). Additionally, no significant changes in expression of other major calcium handling proteins or structural proteins were noted, similar to other transgenic models of *Ryr2* deficiency (19, 21).

Dysfunctional calcium handling is one hallmark of cardiomyopathy and heart failure and a common premise for drug-induced cardiotoxicity. Here, we report that reduced RYR2 protein levels alter calcium handling to show a trend to increased calcium amplitude and reduced relaxation kinetics, matching other transgenic models of reduced Ryr2 expression (21) and diabetic cardiomyopathy (36). As groups/clusters of RYR2 channels function in concert, it is possible that reducing the amount of RYR2 could alter the coupling efficiency between the voltage-gated L-type calcium channel (LTCC) and RYR2, leading to decreased responsiveness to extracellular calcium. As a compensatory mechanism, the cell may increase cytosolic calcium release through a slower RYR2 deactivation (13, 21). Due to an increased reliance of intracellular calcium release for contractility, this may also explain the decreased sensitivity of *RYR2* Het KO cells to verapamil and nifedipine (LTCC blockers). RYR2 channel function is impacted by the cluster size and intra-cluster channel organisation and density. In the future, our model would serve useful for interrogation of this fundamental relationship using live-cell calcium imaging together with super-resolution imaging preferably at the same subset of RYR2 clusters. This would enable a greater understanding of the plasticity and dynamics of excitation-contraction coupling and how differences in RYR2 protein levels impact this at the nanoscale level. Although this is technically challenging there have been recent advances in the field. For example, a transgeneic mouse with a photactivated label on RyR2 has been recently generated (37). To understand the functional differences in basal phenotype and pharmacological response in *RYR2* Het KO vs. WT cardiomyocytes, an unbiased proteomics approach was taken. A limitation of the proteomics was an underpowering of the study due to the reduced replicates of *RYR2* Het KO 2 cells. However, serveral identified proteins were found to be part of a common pathway (pentose phosphate pathway) that is not likely to be by chance and provides support for our approach. In addition, several proteins identified have links to heart disease and were identified having a role in rodent models of *RyR2* haploinsufficiency. One such protein that was downregulated in *RYR2* Het KO 2 cells, CSRP3 (Muscle LIM protein (MLP), was of primary interest because of the literature evidence. CSRP3 is a key regulator of sarcomeric structure with mutations that are linked to hypertrophic and dilated cardiomyopathy development in patients. MLP-deficient mice develop dilated cardiomyopathy and are the first transgenic animal model of heart failure (6). Recently, an *in vitro* human model of *CSRP3* deficiency was reported. *CSRP3* deficient human ES-cell derived cardiomyocytes display defects in calcium handling with increased cytosolic calcium, elevated ROS levels showing a hypertrophic cardiomyopathy phenotype (32). Additionally, CSRP3 has been found to be downregulated in *Ryr2* KO mouse hearts (22). Indeed, knockdown of CSRP3 in the *RYR2* WT isogenic cardiomyocytes caused an increase in beat amplitude, falling time and a reduced sensitivity to verapamil treatment indicating that this protein *per se* contributes to the *RYR2* Het KO phenotype. Sarcolemmal associated membrane protein (SLMAP) was shown to be potentially downregulated in *RYR2* Het KO 2 cells. SLMAP binds to myosin in cardiac muscle, and it is hypothesized to have a potential role in the structural arrangement of excitation coupling apparatus (38). Mutations in SLMAP have been linked to Brugada syndrome with putative deficits in trafficking of the sodium channel Nav1.5 to the cellular membrane (39). SLMAP1 and SLMAP3 transgenic overexpressing mice both show deficits in calcium handling proteins of the sarcoplasmic reticulum including a reduction in RYR2 and or ATP2A2/PLN expression (33, 34). Taken together, the altered expression of CSRP3 and SLMAP that can modulate myosin structure and calcium handling dynamics likely contribute to the *RYR2* Het KO phenotype of calcium handling dysfunction and increased beat amplitude. Conversely, when studying the upregulated proteins in *RYR2* Het KO 2 cells, TGFBI is the second most upregulated protein. Pseudo bulk RNA-sequencing technologies comparing age-associated cell type-specific gene signature in donor and dilated cardiomyopathy hearts revealed that TGFBI positively correlates with aging in DCM cardiomyocytes, but not in normal donors (31). Excitation-contraction coupling is changed with age, and decreased RYR2 expression or post-translational modifications, sarcolemmal dissociation of RYR2 clusters have been found to play a role (40, 41). Additionally, TGFBI has been found as an upstream regulator of mTOR activation in Drosophila models of cardiac disease (42). Here, the most upregulated protein identified was the glucose transporter GTR3 (SLC2A3/GLUT3). GLUT3 is a high-affinity glucose transporter allowing efficient uptake of glucose into the cell and is expressed in the adult human heart (43). *Ryr2* deficient hearts of mice show increased glycolysis (21), and here an upregulation of the pentose phosphate pathway that is also an important part of glucose metabolism. It was found that the pentose phosphate pathway is upregulated in *RYR2* Het KO 2 cardiomyocytes, specifically upregulation of G6PD that is the rate-limiting enzyme of the pentose phosphate pathway. G6PD knockdown in the *RYR2* Het KO 2 cardiomyocytes decreased beat amplitude to a greater magnitude than control siRNA and increased the sensitivity to L-type calcium inhibition with verapamil treatment. In line with our results, G6PD has been shown to interact with the LTCC in rodent cardiac myocytes, and its deficiency alters sensitivity to L-type calcium inhibitors and affects contractile function (44). *RYR2* Het KO 2 cardiomyocytes have an upregulation of the pentose phosphate pathway that may compensate for increased oxidative stress in the cells as a result of increased intracellular calcium. If the cells are already under oxidative stress any treatment with an additional oxidant stressor, such as menadione, would have a greater effect on the redox potential of the cells vs. that of WT. In fact, in response to menadione treatment *RYR2* Het KO cells showed a greater magnitude of reduction in the GSH/GSSG ratio compared to WT cells. Persistent upregulation of the pentose phosphate pathway may exacerbate oxidative damage and contribute to cardiomyopathies (45).

Prior preclinical models of *Ryr2* deficiency have been limited to transgenic animals, with no genetically engineered models to date. Most RYR2 disease models are focused on recapitulating patient characteristics of CPVT (46–48). We focused on creating a generalized model of altered calcium handling dynamics through reduction of RYR2 protein. Typically, in heart disease both the calcium induced calcium release as well as intracellular calcium handling is affected leading to reduced contractility and prolonged relaxation over time. Our model shows a trend to increased calcium amplitude with slower calcium re-uptake. Additionally, activated oxidative stress has been demonsrated in heart failure and our model shows an altered metabolic state with upregulation of the pentose phosphate pathway and increased sensitivity to redox alterations. It may represent a pre-disposed state that requires additional factors for full manifestation of pathophysiological disease. Our model provides proof of concept that pharmacological responses are not the same between healthy and dysfunctional states with altered calcium handling and metabolic disturbances. This supports use of our model and others in safety screening workflows, for example, that typically utilize healthy hiPSC-CM lines. Altogther, this RYR2 deficient model mimics some aspects of heart failure, including calcium handling dysfunction, altered glucose metabolism, differential pharmacological responses, increased sensitivity to redox alterations and allows for the first time, the study of pharmacological functional responses together with the underlying molecular mechanisms as a result of RYR2 deficiency.

## Study limitations

A limitation of the current study is that it may not be informative for investigating potential therapies for specific gain of function pathogenic CPVT variants. However, it may serve useful for testing potential treatments and/or mechanistic investigations for some CPVT-linked RYR2 mutations including deletion of exon-3, G357S point mutation (49, 50) or RYR2 loss of function mutations that result in reduced RYR2 protein levels (51). These cardiomyocytes with decreased RYR2 protein show reduced responsiveness to caffeine and isoproterenol (51). To this end, future experiments to characterize the response of RYR2 Het KO cardiomyocytes to isoproterenol treatment and beta-blockers such as nadolol and propanolol as well as flecainide would be informative. As described in the discussion section the model would serve useful to elucidate reduced RYR2 expression impact on intra-cluster size organization, density on excitation-contraction coupling relevant for loss of function mutations as well as in safety screening workflows. As discussed prior, a limitation in the study is the reduced power of the proteomics to detect differential expression of individual proteins and so emphasis was placed on pathway analysis. Given this limitation we did not observe changes in expression of cardiac stress markers such as NPPA, NPPB, MYH6, MYH7 and ACTA1 proteins nor pathways related to hypertrophy. This observation is also inline with previous rodent models of RyR2 haploinsufficiency that do not report changes in hypertrophy markers (21). These aspects may also be further investigated in future studies of the model.

## Data Availability

The datasets presented in this study can be found in online repositories. The names of the repository/repositories and accession number(s) can be found below: https://www.ebi.ac.uk/pride/archive/, PXD052254 (52).
