# Supplementary material for: RYR2 deficient human model identifies calcium handling and metabolic dysfunction impacting pharmacological responses
Source: Front Cardiovasc Med. 2024 Jul 8;11:1357315. doi: 10.3389/fcvm.2024.1357315 (PMC11260679; doi:10.3389/fcvm.2024.1357315)
Supplement: Supplementary file 1 [file Datasheet1.docx]

Supplementary Material

RYR2 deficient human model identifies calcium handling and metabolic dysfunction impacting pharmacological responses

Linda Starnes^1*^, Andrew Hall^2^, Damla Etal^3^, Anna-Lina Cavallo^3^, Piotr Grabowski^4^, John Gallon^4^, Michelle Kha^1^, Ryan Hicks^5,6^, Amy Pointon^2*^

*** Correspondence:** Linda Starnes, [linda.starnes@astrazeneca.com](mailto:linda.starnes@astrazeneca.com), Amy Pointon, [amy.pointon@astrazeneca.com](mailto:amy.pointon@astrazeneca.com)

# Supplementary Figures and Tables

## Supplementary Figures

**Supplementary Figure 1. (A)** Schematic of guide RNA targeting exon 3 of the *RYR2* gene and the resulting INDELs found in a third heterozygous knockout clone (Het KO 3). **(B)** Table showing the INDELS identified by Amplicon sequencing and the resulting variant at the protein level within the *RYR2* Het KO3 clone. **(C)** Representative immunocytochemistry images of pluripotent marker staining of *RYR2* Het KO 3. Hoechst indicates nuclei, scale bar represents 100 µm (n=3). **(D)** Image of G banding karyotype of *RYR2* Het KO3 hiPSCs (n=20 cells). **(E)** Representative immunocytochemistry images of cardiomyocyte marker (TNNT, ACTN2, ATP2A2, PLN) staining of *RYR2* Het KO 3. Hoechst indicates nuclei, scale bar represents 100 µm (n=3). **(F)** Average percent positive cells for cardiac troponin T expression measured by flow cytometric analysis at day 25 of differentiation in WT, *RYR2* Het KO 3 hiPSC-derived cardiomyocytes (hiPSC)-CMs. Mean ± SEM (n=3) **(G)** Representative western blot of RYR2 Het KO 3 hiPSC-CMs. **(H)** Impedance-based measurements of paced 1 Hz beat rate and **(I)** beat amplitude of WT and *RYR2* Het KO3 differentiated cardiomyocytes at day 25 of differentiation (n=4). Statistics in figures are performed by unpaired two-tailed t-test; *** p-value<0.001.


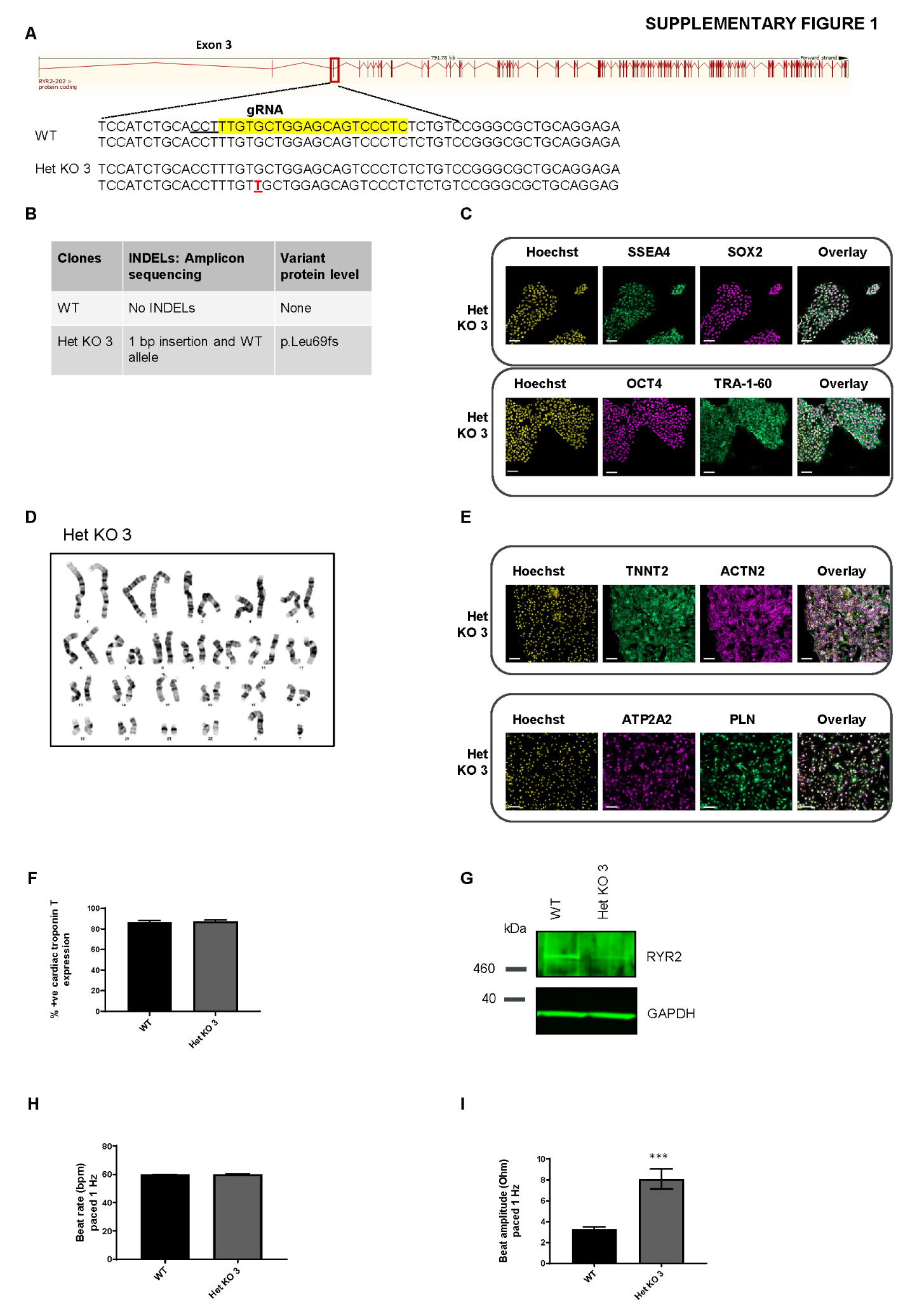

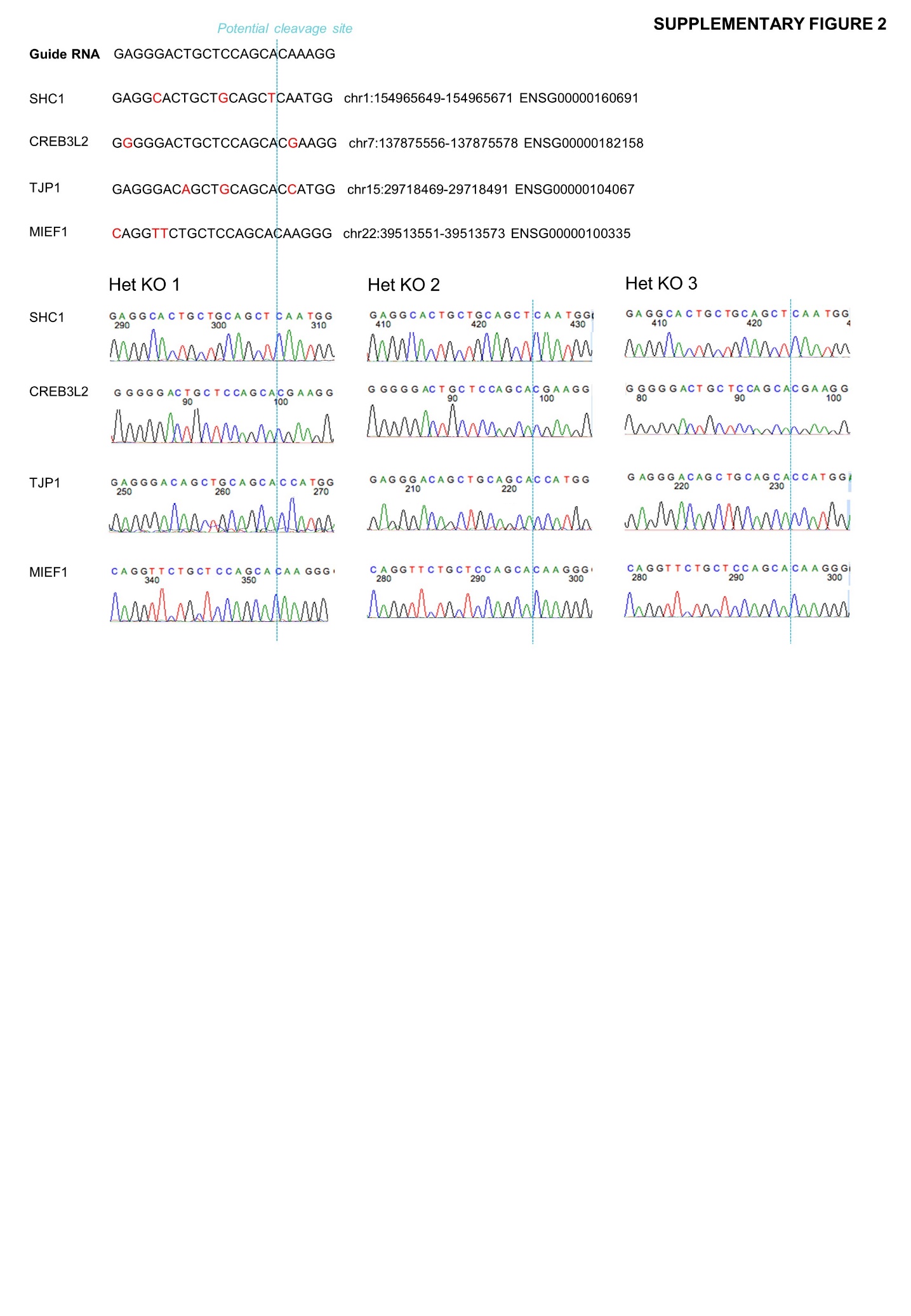


**Supplementary Figure 2.** Potential off-target sites were identified as known coding or regulatory sequences where the gRNAs had full PAM site complementarity and/or fewer than 5 mismatches within the target. Shown are the chromatogram synopses from PCR genotyping that showed no evidence for off target events.



**Supplementary Figure 3.** Representative calcium transients of RYR2 Het KO 2 and WT cardiomyocytes showing that Het KO cardiomyocytes have a larger amplitude, and longer fall time, compared to WT cardiomyocytes. Calcium transients (Fluo4 fluorescence) were imaged using a Zeiss LSM 800 Confocal microscope. Fluo4 fluorescence was stimulated by 488 nm laser, and emission captured between 510 and 550 nm with an image taken every  250 ms. Calcium transients were analyzed using ImageJ.


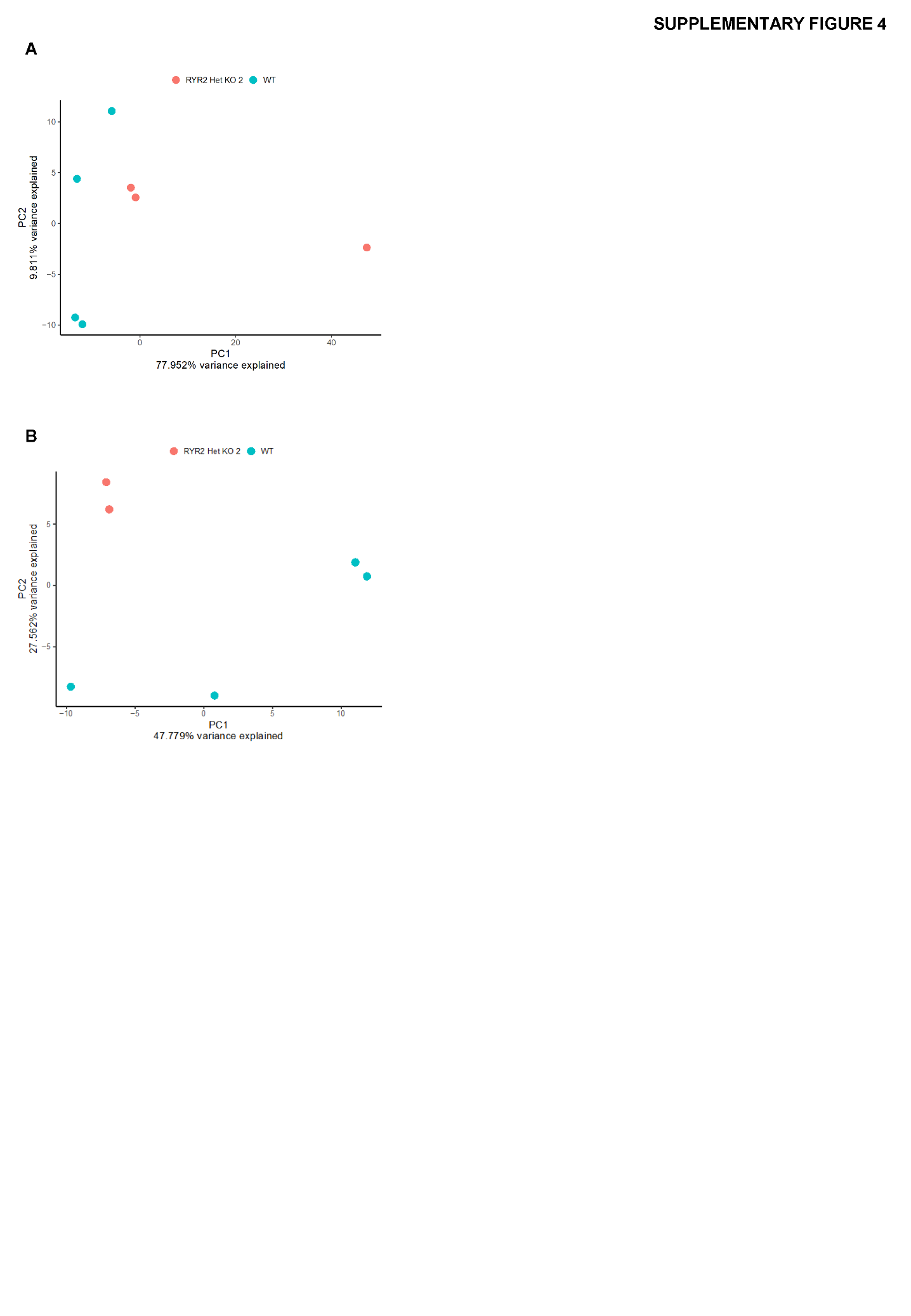


**Supplementary Figure 4.** **(A)** Principal component analysis of 7 cardiomyocyte samples (4 WT and 3 Het KO 2) based on normalised LFQ expression values of all proteins present in the filtered dataset (4,907). **(B)** Principal component analysis of samples after suspected Het KO 2 outlier removal that were used in the differential protein expression analysis.
